# Anti-Interferon Autoantibodies in Autoimmune Polyendocrinopathy Syndrome Type 1

**DOI:** 10.1371/journal.pmed.0030289

**Published:** 2006-06-13

**Authors:** Anthony Meager, Kumuthini Visvalingam, Pärt Peterson, Kaidi Möll, Astrid Murumägi, Kai Krohn, Petra Eskelin, Jaakko Perheentupa, Eystein Husebye, Yoshihisa Kadota, Nick Willcox

**Affiliations:** **1**Biotherapeutics, National Institute for Biological Standards and Control, South Mimms, United Kingdom; **2**Molecular Pathology, Institute of General and Molecular Pathology, Biomedicum, University of Tartu, Tartu, Estonia; **3**Institute of Medical Technology, University of Tampere, Tampere, Finland; **4**Department of Human Molecular Genetics, National Public Health Institute, Helsinki, Finland; **5**Hospital for Children and Adolescents, University of Helsinki, Helsinki, Finland; **6**Division of Endocrinology, Institute of Medicine, University of Bergen, Norway; **7**Department of Medicine, Haukeland University Hospital, Bergen, Norway; **8**Neurosciences Group, Weatherall Institute of Molecular Medicine, University of Oxford, Oxford, United Kingdom; University of OsloNorway

## Abstract

**Background:**

The *autoimmune regulator (AIRE)* gene influences thymic self-tolerance induction. In autoimmune polyendocrinopathy syndrome type 1 (APS1; OMIM 240300), recessive *AIRE* mutations lead to autoimmunity targetting endocrine and other epithelial tissues, although chronic candidiasis usually appears first. Autoimmunity and chronic candidiasis can associate with thymomas as well. Patients with these tumours frequently also have high titre immunoglobulin G autoantibodies neutralising type I interferon (IFN)–α and IFN-ω, which are secreted signalling proteins of the cytokine superfamily involved in both innate and adaptive immunity.

**Methods and Findings:**

We tested for serum autoantibodies to type I IFNs and other immunoregulatory cytokines using specific binding and neutralisation assays. Unexpectedly, in 60/60 Finnish and 16/16 Norwegian APS1 patients with both *AIRE* alleles mutated, we found high titre neutralising immunoglobulin G autoantibodies to most IFN-α subtypes and especially IFN-ω (60% homologous to IFN-α)—mostly in the earliest samples. We found lower titres against IFN-β (30% homologous to IFN-α) in 23% of patients; two-thirds of these (from Finland only) also had low titres against the distantly related “type III IFN” (IFN-λ1; alias interleukin-29). However, autoantibodies to the unrelated type II IFN, IFN-γ, and other immunoregulatory cytokines, such as interleukin-10 and interleukin-12, were much rarer and did not neutralise.

Neutralising titres against type I IFNs averaged even higher in patients with APS1 than in patients with thymomas. Anti–type I IFN autoantibodies preceded overt candidiasis (and several of the autoimmune disorders) in the informative patients, and persisted for decades thereafter. They were undetectable in unaffected heterozygous relatives of APS1 probands (except for low titres against IFN-λ1), in APS2 patients, and in isolated cases of the endocrine diseases most typical of APS1, so they appear to be APS1-specific.

Looking for potentially autoimmunising cell types, we found numerous IFN-α^+^ antigen-presenting cells—plus strong evidence of local IFN secretion—in the normal thymic medulla (where *AIRE* expression is strongest), and also in normal germinal centres, where it could perpetuate these autoantibody responses once initiated. IFN-α2 and IFN-α8 transcripts were also more abundant in antigen-presenting cells cultured from an APS1 patient's blood than from age-matched healthy controls.

**Conclusions:**

These apparently spontaneous autoantibody responses to IFNs, particularly IFN-α and IFN-ω, segregate like a recessive trait; their high “penetrance” is especially remarkable for such a variable condition. Their apparent restriction to APS1 patients implies practical value in the clinic, e.g., in diagnosing unusual or prodromal *AIRE*-mutant patients with only single components of APS1, and possibly in prognosis if they prove to predict its onset. These autoantibody responses also raise numerous questions, e.g., about the rarity of other infections in APS1. Moreover, there must also be clues to autoimmunising mechanisms/cell types in the hierarchy of preferences for IFN-ω, IFN-α8, IFN-α2, and IFN-β and IFN-λ1.

## Abstract 2

Almost all of nearly 100 APS1 patients studied made large amounts of auto-antibodies that blocked the function of IFN-α and IFN-ω. The antibodies appeared early during development of the disease and may play a role in its etiology.

## Editors' Summary

**Background.:**

The human body is under constant attack by viruses, bacteria, fungi, and parasites, but the immune system usually prevents these pathogens from causing disease. To be effective, the immune system has to respond rapidly to foreign antigens (bits of protein specific to pathogens) while ignoring self-antigens. If tolerance to self-antigens breaks down, autoimmunity develops, often causing disease. There are many common autoimmune diseases—type I diabetes and multiple sclerosis, for example—but because these involve defects in many genes as well as environmental factors, the details of how autoimmunity develops remain unclear. Autoimmune polyendocrinopathy syndrome type 1 (APS1), however, is caused by defects in a single gene. Patients with this rare disease characteristically have defects (or mutations) in both copies of a gene called *AIRE* (for autoimmune regulator). In normal people, the protein product of this gene helps to establish tolerance to a subset of self-antigens. People carrying *AIRE* mutations make an autoimmune response against some of their own tissues, typically the endocrine (hormone-producing) tissues that control body metabolism. A major component of this autoimmune response are “autoantibodies” (antibodies are immune molecules that normally recognize and attack foreign substances, whereas autoantibodies are directed against the body's own molecules).

**Why Was This Study Done?:**

For a diagnosis of APS1, a patient must have at least two of the following symptoms: recurrent, localized yeast infections (usually the first symptom of the disease to appear in early childhood), hypoparathyroidism (failure of the gland that controls calcium levels in the body), and Addison disease (failure of the steroid-producing adrenal glands, which help the body respond to stress). The researchers who did this study had previously noticed that these yeast infections and autoimmunity (usually against muscle) can also occur in patients with tumors of the thymus (thymomas). The thymus is the organ that generates immune cells called T cells. Generation of the T cell repertoire in the thymus involves selection of those T cells that recognize only foreign substances. T cells that can react against self-antigens are eliminated, and the *AIRE* gene is thought to be involved in this “education process.” Like those with APS1, patients with thymomas make autoantibodies not only against target organs (especially muscle in their case), but also against interferon alpha (IFN-α) and interferon omega (IFN-ω), two secreted immune regulators. The researchers wanted to know if patients with APS1 also make autoantibodies against interferons, because this could provide insights into how autoimmunity develops in APS1 and other autoimmune diseases.

**What Did the Researchers Do and Find?:**

The researchers tested blood from nearly 100 APS1 patients for antibodies to IFN-α, IFN-ω, and other immunoregulatory cytokines. They found that almost all patients made large amounts of antibodies that blocked the function of IFN-α and IFN-ω; some also made lower amounts of antibodies against two related interferons, but none made blocking antibodies against unrelated interferons or other immune regulators. For many patients, serum samples were available at different times during their disease, which allowed the researchers to show that the antibodies appeared early in disease development, before the onset of yeast infections or damage to endocrine tissues, and their production continued for decades as the patient aged. Furthermore, only patients with APS1 made these antibodies—they were absent in patients with Addison disease alone, for example.

**What Do These Findings Mean?:**

The discovery that autoantibodies to IFN-α and IFN-ω are made persistently in patients with APS1 suggests ways in which autoimmunity develops in these patients. These can now be investigated further both in patients and in animal models of the disease. The discovery also has practical implications. Measurement of these autoantibodies might help some APS1 patients by allowing earlier diagnosis—and prompter treatment—than in current practice. The levels of these autoantibodies might also help to predict the time course of APS1 in individual patients, although more studies will be needed to check this out. Finally, if future studies show that interferon autoantibodies are responsible for the patients' susceptibility to yeast infections (which seems plausible), treatment with IFN-γ, an interferon to which APS1 patients do not make autoantibodies, might provide an alternative way to deal with this problem.

**Additional Information.:**

Please access these Web sites via the online version of this summary at http://dx.doi.org/10.1371/journal.pmed.0030289.

• MedlinePlus pages on autoimmune diseases

• Online Mendelian Inheritance in Man page on APS1

• Links to patient information on APS1 from the Stanford Health Library

• Wikipedia page on autoendocrine polyendocrinopathy (note: Wikipedia is a free online encyclopedia that anyone can edit)

• Information on autoimmunity from the American Autoimmune Related Diseases Association

## Introduction

The *autoimmune regulator (AIRE)* gene encodes a two-plant-homeodomain zinc-finger protein that acts as a transcriptional regulator [1–4]. *AIRE* is highly expressed in thymic medullary epithelial cells (MECs), where it is strongly implicated in the expression of some peripheral self-antigens and the induction of tolerance thereto [1–6]; it is also expressed in dendritic cells (DCs) [5], which are very potent antigen-presenting cells (APCs). Over 50 recessive mutations have been identified in the coding region of *AIRE*. In homozygotes or compound heterozygotes, they cause autoimmune polyendocrinopathy syndrome type 1 (APS1; OMIM 240300), also known as autoimmune polyendocrinopathy candidiasis ectodermal dystrophy [2–4,6–8]. However, in at least 2% of clinically typical patients, no mutations are found [8].

Probably through defects in AIRE protein function, in self-antigen expression by MECs, and in self-tolerance induction in autoreactive T cells as they are generated in the thymus, the ensuing autoimmune attack focusses particularly on epidermal and endocrine tissues, and features both T cells and autoantibodies [1–4,7,8]. By definition, the patients have at least two of the “APS1 triad”—hypoparathyroidism (HP), Addison disease (AD), and chronic muco-cutaneous candidiasis (CMC). However, the clinical phenotype is highly variable. Characteristically, patients present with CMC and/or skin disorders, usually early in childhood; these symptoms are followed (sometimes 1–3 decades later) by autoimmune endocrine disorders, which may also target the gonads and/or endocrine cells in the gut, pancreatic islets, and thyroid gland [3,4,7,8]. The candidiasis, which is evident early in nearly all APS1 patients, may be due to defects in *AIRE* signalling pathways involved in the handling of Candida sp. infection.

Interestingly, CMC also co-occurs with autoimmunity (albeit against different targets) in occasional patients with thymomas [9]. We have recently found—at diagnosis—high titre immunoglobulin G (IgG) neutralising autoantibodies to interferon (IFN)–α (all 12 subtypes) and IFN-ω, and to interleukin (IL)–12, in many patients with thymoma, with late-onset myasthenia gravis (MG), and especially with thymoma and MG together [10,11]. The human type I IFNs all use the same receptor, and fall into five classes: IFN-α, IFN-β, IFN-ɛ, IFN-κ, and IFN-ω [12]. Only the IFN-α group contains multiple closely related “subtypes”, to which IFN-ω and IFN-β are about 60% and 30% related, respectively [12]. IFN-α, IFN-β, and IFN-ω are inducibly expressed at very high levels in plasmacytoid DCs and monocytes following viral infection or stimulation through Toll-like receptors [12,13], and are key early players in innate immune responses. In addition, both type I IFNs and IL-12 polarise towards pro-inflammatory T helper 1 responses in humans [14,15], and perhaps less so within MG thymomas [16].

The recently identified human “type III IFNs”, IFN-λ1, IFN-λ2, and IFN-λ3 (alias IL-29, IL-28A, and IL-28B, respectively), are related structurally to, and share ∼15% homology with, type I IFNs [17,18]. They act through a distinct receptor comprising a unique IFNLR subunit and an IL-10Rβ subunit (also part of the IL-10, IL-22, and IL-26 receptors) [17–19]. IL-10 itself is 11%–13% homologous to the type III IFNs [17]. Like the type I IFNs, the IFN-λs are expressed by human peripheral blood mononuclear cells and plasmacytoid DCs upon infection with viruses or stimulation with double-stranded RNA [17,18]. The single type II IFN, IFN-γ, shows essentially no homology to type I or III IFNs [12], and is produced by T cells and natural killer cells.

Autoimmunising processes are very hard to study in humans: there must be valuable clues in the thymomas, in which we find evidence for immunisation against IFN-α and IL-12 [20,21]. This autoimmunisation must be highly selective, since we have rarely found autoantibodies against many other cytokines—even including IFN-β [10]—and never against IFN-γ [11]. Indeed, all of these autoantibodies are also rare in many other infectious, neoplastic, or autoimmune diseases, including sporadic multiple sclerosis and, importantly for the present study, thyroid disease and type 1 diabetes mellitus [11].

In view of these parallels with thymoma patients, we tested for similar anti-cytokine autoantibodies in APS1 patients (and controls); though generally rare, the disease is less uncommon in Finnish (1:25,000), Sardinian (1:14,400), Iranian Jewish (1:9,000), and Norwegian (1:80,000) populations [4,8]. We therefore screened coded serum samples from a well-studied Finnish cohort [8], and from a further smaller group of Norwegian APS1 patients, in validated binding enzyme-linked immunoabsorbent assays (ELISAs) and neutralising bioassays against type I, II, and III IFNs, IL-10, and IL-12 [10,11]. We focussed on the genetics, specificity, kinetics, and clinical correlates of the anti-IFN autoantibodies we found, and also tried to locate potentially autoimmunising cell types in normal thymus and reactive tonsils.

## Methods

### Patients

We studied the 77 Finnish APS1 patients [8] from whom sera were available (initially taken at ages 3.3 to 57 y [mean 19.6]). We tested 64 patients who had been *AIRE*-genotyped (see Table 1) and another 13 “untyped” but clinically typical APS1 patients. From 51 of the *AIRE*-genotyped patients, we tested 1–5 further samples dating from 0.7 to 33 y later (mean 16). The diagnosis of APS1 was based on the classical clinical criteria (presence of at least two of CMC, HP, and AD). The patients had from one to eight (median four) autoimmune manifestations, with onset from 0.7 to 20 y later (mean 6.7). Almost all had had oral or nail candidiasis or both, since the age of 0.2 to 30 y. Their endocrine disorders are summarised in Table 2; in addition, 22 had alopecia (since ages 3.8–30 y), 16 had keratoconjunctivitis (1.3–16), 16 had vitiligo (0.7– 45), six had had a period of vasculitis, and two were growth-hormone-deficient.

**Table 1:**
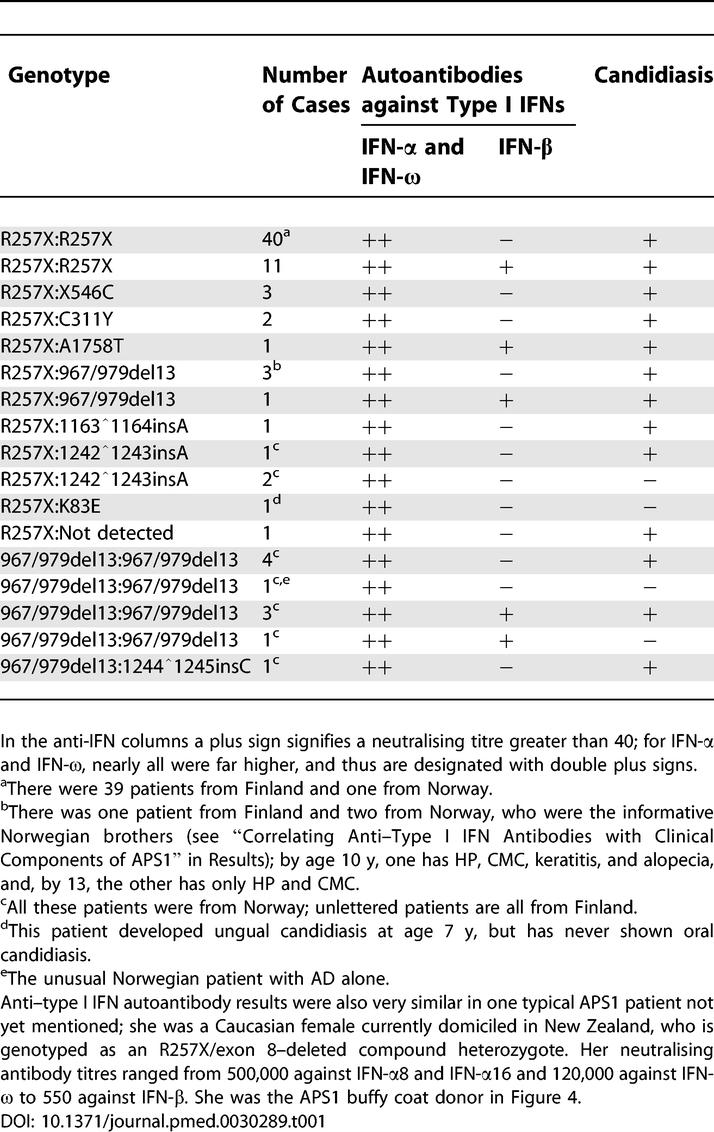
Anti–Type I IFN Antibodies, *AIRE* Genotypes, and Candidiasis in APS1 Cases from Finland and Norway

**Table 2:**
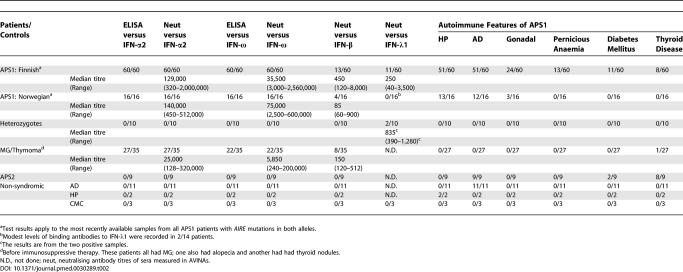
Binding and Neutralising Anti-IFN Autoantibodies in APS1 Patients with *AIRE* Mutations, in their Unaffected Heterozygous Relatives, and in Patients with MG/Thymoma (as a “Positive” Control Group), with APS2, or with Sporadic Diseases from the APS1 Triad

We also studied 17 genotyped APS1 or APS1-like patients from Norway (16 of Norwegian and one of Pakistani origin) [22]. The majority had at least two of the APS1 triad (see Table 2 for endocrine disorders and Table 1 for CMC), although one male aged 35 y had only AD ([22]; see footnote “e” of Table 1). Two other informative brothers in this cohort (see footnote “b” of Table 1) have not yet developed AD by ages 10 and 13 y [23].

As healthy and disease controls, we tested sera from ten *AIRE*-heterozygous unaffected first-degree relatives (six from Finland with R257X allele and four from Norway—two with R257X, one with 1242̂1243insA, and one with 967/979del13 alleles) and from patients with APS2 (*n =* 9) or sporadic AD (*n =* 11), HP (*n =* 2), or CMC (*n =* 3)—all without APS1. Six patients with sporadic AD and eight patients with APS2 were found negative for the two most common APS1 *AIRE* mutations, R257X and 967/979del13.

The patients with MG and thymoma have been reported on in detail previously [10,11]; we summarise their results here purely for comparison. All samples were taken with informed consent and local ethical committee approval; the samples were coded and stored at −20 °C and were diluted in the media appropriate for cytokine-specific immunoassays and bioassays (see below).

### IFNs and Other Cytokines

IFNs and other cytokines, generously donated by the stated manufacturers, were used in autoantibody-binding ELISA and/or antiviral interferon neutralisation assay (AVINA) specific for the following: recombinant human IFN-α2a (Hoffmann-La Roche, Basel, Switzerland); other IFN-α subtypes (BPL Laboratories, Piscataway, New Jersey, United States); IFN-β (Biogen, Boston, Massachusetts, United States); IFN-ω (Bender and Co., Vienna, Austria); IFN-γ (Roussel-Uclaf, Romainville, France); granulocyte-macrophage colony-stimulating factor (Immunex, Seattle, Washington, United States); IFN-λ1 [IL-29], IFN-λ2 [IL-28A], and IL-4 (R&D Systems Minneapolis, Minnesota, United States); IL-10 (Schering-Plough, Kenilworth, New Jersey, United States); IL-12 (Hoffman-La Roche); and TNF-α (Genentech, South San Francisco, California, United States).

### Binding ELISA for the Detection of Anti-IFN and Anti-Cytokine Autoantibodies

Microtitre wells (Dynatech, Orlando, Florida, United States) were coated with IFN or cytokine solutions at 2 μg protein/ml (PBS [pH 7.0]), 0.1 ml per well, for 2 h at 22 °C. Wells were blocked with 3.0% HSA in PBS for 0.5 h at 22 °C or overnight at 4 °C, and serially diluted sera were added for 2 h at 22 °C, before washing and development with anti-human IgG (γ-chain-specific)–alkaline phosphatase conjugate (Sigma Chemical, St. Louis, Missouri, United States; 0.1 ml/well of 1:1,000 dilution at 22 °C for 2 h), further washing, addition of p-nitrophenyl phosphate substrate for 0.5 h at 22 °C, and then of 3M sodium hydroxide (0.05 ml/well); absorbances were read at 405 nm. We considered as positive any values greater than two standard deviations above the mean of controls.

### AVINA

For type I IFNs, we pretreated the human glioblastoma cell line 2D9 (generously provided by Dr. W. Däubener; [24]) with dilute IFN preparations (ten laboratory units [LU] per millilitre) that had been pre-incubated for 2 h with serial dilutions of test sera [25,26]. For IFN-λs, instead of 2D9, we used the human glioblastoma cell line LN319 (generously provided by Dr. A.-C. Diserens [27]), which responds well to their antiviral activity [28]. The cells were then challenged with encephalomyocarditis virus for 24 h, stained with 0.05% amido blue black, fixed with 4% formaldehyde in acetic acid buffer, and stain-eluted with 0.15 ml of 0.05 M sodium hydroxide solution before absorbance was read at 620 nm. The neutralising antibody titre [29] was the dilution of serum that reduces 10 LU/ml of IFN to 1 LU/ml (the normal end point of antiviral assays [25]). The cut-off for positivity was a titre of 40, which numerous healthy controls never exceeded. We routinely included IFN-α1 (alias IFN-α13); its low specific activity consistently results in ∼10-fold lower neutralising antibody titres than for any other IFN-α subtype. While these titres generally correlate well with those for IFN-α2, they have been omitted to avoid creating a misleading impression.

### Immunohistology

Paraffin sections of tonsils or normal child thymus were de-waxed, heated under pressure for 2 min in Tris:EDTA buffer (pH 9.0), and washed in PBS. They were then stained for 1 h at 20 °C with the anti-MxA monoclonal antibody M143 (1:200 [30]), and then with sheep anti-IFN-α (1:1,000 [31,32]) for 30 min before washing and incubation with donkey anti-mouse IgG-Alexa 594 (1:600) and anti-sheep IgG-Alexa 488 (1:400; both from Molecular Probes, Invitrogen, Carlsbad, California, United States), before washing and mounting.

### Generation of Monocyte-Derived DCs

Buffy coats were collected from an APS1 patient with an R257X mutation in one *AIRE* allele and 967/979del13 in the other, and from two healthy blood donors; the buffy coats were washed and then cryopreserved in liquid nitrogen. Cells were then thawed and cultured in RPMI 1640/10% fetal calf serum growth medium for 72 h at 37 °C in a 5% carbon dioxide incubator. Monocytes were enriched with monoclonal anti-CD14-antibody-coated microbeads (Miltenyi Biotec, Bergisch Gladbach, Germany), and cultured for seven further days with IL-4 (1,000 U/ml); granulocyte-macrophage colony-stimulating factor (800 U/ml) was added on days 0, 3, and 6 to yield “immature DCs”. On day 7, some of the DCs were matured for 3 d with monocyte-conditioned medium prepared as previously described [33].

### Quantitative Real-Time PCR

RNA was isolated using TRIzol (Life Technologies, Gaithersburg, Maryland, United States) and reverse-transcribed to cDNA using the First-Strand cDNA Synthesis kit (Fermentas, Burlington, Ontario, Canada). Quantitative real-time PCR (RT-PCR) was performed with the ABI Prism 7000 SDS instrument (Applied Biosystems, Foster City, California, United States) using qPCR SYBR Green Core Kit (Eurogentec, Seraing, Belgium) according to the manufacturer's instructions, except that a 2 mM magnesium chloride concentration was used. The amplification program included an initial denaturation step at 95 °C for 10 min, followed by denaturation at 95 °C for 15 s, and annealing and extension at 60 °C for 1 min, for 50 cycles. SYBR Green fluorescence was measured after each extension step, and the specificity of amplification subjected to melting curve analysis. As an internal reference for quantification experiments, we performed RT-PCR reactions with the house-keeping genes *GAPDH* and *HPRT*. The primers were as follows: *GAPDH* forward, tccaccaccctgttgctgtag; *GAPDH* reverse, gaccacagtccatgccatcact; *HPRT* exon 6, gactttgctttccttggtcagg; *HPRT* exon 7, agtctggcttatatccaacacttcg; *IFN-ω* forward, gaggtacttccagggaatccg; *IFN-ω* reverse, catttcaagatgagcccaggtc; *IFN-α2* forward, cctgatgaaggactccatt; *IFN-α2* reverse, aaaaaggtgagctggcatacg; *IFN-α8* forward, gtgatagagtctcccctgatgtac; and *IFN-α8* reverse, cttcaatcttttttgcaagttga [34].

## Results

### Autoantibodies against IFN-α and IFN-ω

We first screened for anti-IFN autoantibodies in 60 typical Finnish APS1 patients with both *AIRE* alleles mutated, and then in 16 such patients from Norway (Table 2). Both binding and neutralising autoantibodies against IFN-α2 and IFN-ω were readily detected in the last samples from all 76 patients, regardless of their exact clinical phenotype. These autoantibodies were clearly IgG, as a specific anti-human γ-chain conjugate was used to quantify them in the ELISA. In AVINAs, titres against IFN-α2 and IFN-ω were greater than 5,000 in nearly all samples. They were generally 3- to 6-fold higher than in MG/thymoma cases (Table 2). Since the ten unaffected heterozygous first-degree relatives of APS1 patients were uniformly negative in both assays (Table 2), this response evidently segregated like a recessive trait. Moreover, it appears to be APS1-restricted (see below). In our previous study [11], no neutralising autoantibodies to either IFN-α2 or IFN-ω were detected in sera from 70 healthy controls.

These findings raise numerous questions about this anti-IFN response in APS1 patients that we now address; they concern its kinetics, its specificity for different IFN-α subtypes, other IFNs, and other cytokines, its specificity for APS1 versus its component diseases, its correlation with the various *AIRE* genotypes, and the mechanisms of autoimmunisation.

#### Kinetics of anti-IFN-α and anti-IFN-ω autoantibodies.

**Figure 1:**
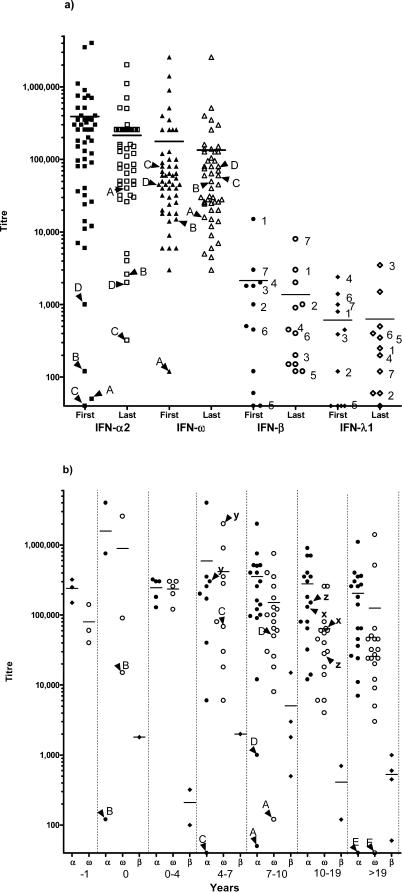
Anti-IFN Neutralisation Titres in APS1 Patients at Different Sampling Times (A) Anti-IFN neutralisation titres in the first and last available serum samples from 51 *AIRE*-genotyped Finnish patients. We use arrowheads to mark the unusual APS1 patients A–D (detailed in Table 3), and numerals for patients whose samples neutralised both IFN-β and IFN-λ1. Bars indicate geometric mean titres. From first to last available samples (average interval = 16 y), titres were substantially increased, unchanged, or decreased in 19%, 43%, and 38% patients, respectively. (B) Anti-IFN neutralisation titres in Finnish APS1 patients against IFN-α2, IFN-ω, and IFN-β in the first available sera in relation to the time of diagnosis of CMC. Results are grouped for patients sampled 1 yr prior to diagnosis of CMC (−1), at diagnosis (0), or within the indicated number of years thereafter (0–4, 4–7, 7–10, 10–19, or >19 y). This figure excludes four otherwise typical APS1 patients who never developed overt CMC but who also had high titres against IFN-ω (see text); it includes 63 *AIRE*-genotyped and 11 untyped patients. For IFN-β, only the positive patients are shown (for clarity). Arrowheads mark unusual APS1 patients: patients A–E (detailed in Table 3); patient x, who currently has only one detectable *AIRE* mutation; patients y and z, who are two of the three Finnish patients with no detectable *AIRE* mutations; and patient E, who meets the criteria for APS1 (Table 3), but for whom no neutralising autoantibodies or *AIRE* mutations have yet been found.

Serial samples were available from 51 Finnish patients (2–6 from each). Neutralising titres against IFN-ω were already high in the first samples from 50 of these (Figure 1A), and tended to be higher in the younger patients (though not significantly). We have no explanation for their later appearance in the remaining patient (patient A; Table 3).

**Table 3:**
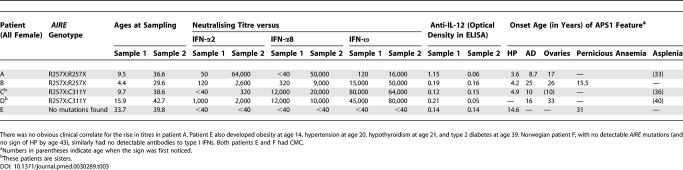
Autoantibodies against IFN-α2, IFN-α8, IFN-ω, and IL-12 and Autoimmune Components in Five “Unusual” Finnish APS1 Patients

Titres were even higher against IFN-α2 than IFN-ω (1.6-fold on average) in 47 of these 51 patients, extending up to 4 × 10^6^ in one 3.3-y-old patient (Figure 1A); in another three of these 51 patients (patients B–D), titres were initially high against IFN-ω (>10,000; Table 3; see below), even when they were low or undetectable against IFN-α2. Notably, in all patients tested, neutralising antibody titres remained high for all subsequent time points up to 30 y thereafter (Figure 1A).

#### Preferences for IFN-α subtypes and IFN-ω.

**Figure 2:**
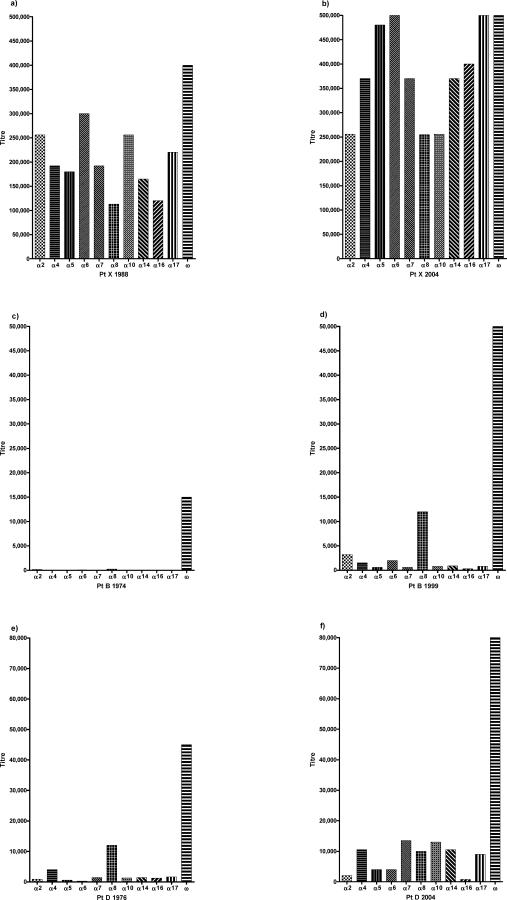
Typical and Unusual Neutralisation Profiles against IFN-α Subtypes and IFN-ω Patient (Pt) X, a Finnish R257X homozygote, has a typical profile (A and B); patients B (C and D) and D (E and F) have unusual profiles (also detailed in Table 3).

When we tested against a panel of ten IFN-α subtypes, sequential samples from 30/30 randomly chosen Finnish patients strongly neutralised all of the IFN-αs as well as IFN-ω (Figure 2A and 2B show a typical example). They also neutralised “leukocyte IFN preparations” produced by virally infected human peripheral blood mononuclear cells in bulk (unpublished data); these heterogeneous mixtures of IFN-α subtypes plus IFN-ω are representative of the IFNs produced during viral infections of leukocytes [35]. Together, these results argue strongly that the antibodies are recognising the native molecules.

In general, neutralisation profiles (and titres) were consistent between the first and last available samples from each patient (Figure 2A and 2B). However, there was an initial preference for IFN-ω (and/or IFN-α8) in three of the 51 patients (patients B–D; Figure 1A; Table 3) that broadened over the following ∼25 y to affect other IFN-α subtypes in patient D (Figure 2E and 2F) and particularly IFN-α8 in patient B (Table 3; Figure 2C and 2D). We can see no obvious clinical explanation for the markedly higher titres in these later samples (Table 3).

#### Correlations with *AIRE* mutations and human leukocyte antigen genotypes.

All 60 genotyped APS1 Finnish patients with mutations in both *AIRE* alleles had at least one R257X allele—which is the predominant Finnish mutation [4,8]—whereas 12 of the 16 patients from Norway had at least one 967/979del13 mutation (Table 1). All the homozygotes—and all the compound heterozygotes—had high titres against IFN-α2 and IFN-ω (Table 1). There may be other defects in the AIRE or parallel pathways, as occasionally clinically typical APS1 patients have only one identified *AIRE* mutation or none at all [8]. Titres were high in the one patient with only one identified *AIRE* mutation (Table 1; “x” in Figure 1B) and even in two of the four individuals with no detected *AIRE* mutations (“y” and “z” in Figure 1B); the other two were completely negative against all IFNs tested, but neither had the full APS1 triad by age 39 (patients E and F; see footnote of Table 3) and one (patient F) is the only affected member of a sibship of 14. Titres were also high in a further 13 “untyped” Finnish APS1 patients (included in Figure 1B).

Both anti-IFN titres and HLA-DR and -DQ types were available for 61 Finnish APS1 patients. HLA-DR15 was somewhat over-represented among the cases with very high titres (>256,000), though not significantly so after correction for multiple comparisons.

#### Correlating anti–type I IFN antibodies with clinical components of APS1.

Some patients were strongly positive for anti-IFN antibodies despite never manifesting the full APS1 triad of clinical features (AD, HP, and CMC; Table 2), including one Norwegian patient who had only AD (see footnote “e” of Table 1). Similarly, titres were already high against IFN-α2 and IFN-ω at ages 3 and 7 y in two highly informative Norwegian brothers with APS1 but no AD (see footnote “b” of Table 1). At that time, both were negative for all ten of the autoantibodies most commonly associated with APS1 [23]; only in their next samples (∼5 y later) did we detect any of these (and then only against 21-hydroxylase [in both brothers] and against aromatic L-amino-acid decarboxylase [in the elder]; E. Husebye, unpublished data). A few other patients were first sampled—and already had high anti-IFN titres—before they developed the full APS1 triad. For example, AD began 6 and 21 y later in two Finnish patients, and HP began 2.7 and 12 y later in two others; again, the relevant endocrine autoantibodies probably appeared after a considerable delay (they are usually found only shortly before the AD [36]).

Moreover, the anti-IFN-α and anti-IFN-ω antibodies were clearly not an effect of CMC; in all the informative cases, the antibodies antedated or coincided with the diagnosis of CMC (Figure 1B), and they also reached high titres in the five patients who never developed CMC overtly (one was a K85E compound heterozygote; see footnote “d” of Table 1).

These antibodies are apparently restricted to APS1. They did not reach significant levels (>1/40) in patients with APS2 (*n =* 9), who show characteristic adult-onset AD, thyroid disease, and/or insulin-dependent diabetes without CMC or HP [37], nor in patients with sporadic AD (*n =* 11), HP (*n =* 2), or CMC (*n =* 3), all without APS1 (Table 2).

### Autoantibodies against Other Interferons and Cytokines

Surprisingly, other infections are rarely problematic in APS1 [8], but we nevertheless screened against other cytokines.

#### IFN-β.

As in patients with both MG and thymoma [10,11], we detected antibodies against the more distantly related type I IFN, IFN-β (30% homology with IFN-α), much less frequently than against either IFN-α or IFN-ω, and at much lower titres (Tables 1 and 2; Figure 1A); we found them mostly in patients with especially high titres against IFN-α2 and IFN-ω (*n =* 15), and usually from the first sample onwards. When positive, titres against IFN-β (in the Finnish cohort) averaged slightly higher than in MG/thymoma patients (Table 2). They were consistently negative in the ten unaffected heterozygotes, and in patients with APS2 (*n =* 9) or sporadic cases of AD alone (*n =* 11), CMC alone (*n =* 3), or HP alone (*n =* 2) (Table 2).

#### IFN-λs.

These recently identified “type III” IFNs are ∼15% homologous to the type I IFNs [17,18]. As yet, there have been no reports of autoantibodies to them; indeed, we found none in the Norwegian cohort. However, we saw clear neutralisation of IFN-λ1 in about two-thirds of the Finnish APS1 sera with anti-IFN-β antibodies but in very few of the others (Table 2; numerals in Figure 1A); the 11 positive patients were all *AIRE* R257X homozygotes. These autoantibodies were not always constant, either disappearing (*n =* 1) or appearing de novo (*n =* 3) several years after anti-IFN-α or anti-IFN-ω autoantibodies were first detected. These results argue against cross-neutralisation of IFN-β and IFN-λ1 by the same antibodies. So do the independent fluctuations in titres against IFN-β and IFN-λ1 in patients 3 and 7 (Figure 1A). Neutralisation of IFN-λ2 was found only in the samples positive against IFN-λ1, and generally at lower titres (A. Meager, unpublished data).

Notably, two of the ten unaffected heterozygotes also had neutralising antibodies against IFN-λ1 (at ages 32 and 39 y; Table 2) but not detectably against IFN-λ2. However, we detected none of these antibodies in sera from the patients with sporadic CMC alone (*n =* 3) or HP alone (*n =* 2) (Table 2) or from 18 healthy controls. In MG/thymoma patients, these autoantibodies were rare and very low in titre, even in sera positive against IFN-β (unpublished data).

#### Other cytokines.

We tested against other cytokines only in the 77 Finnish sera. Against IFN-γ, only “late” sera from two R257X homozygous patients (one from ∼28 and 37 y and the other ∼17 y after onset) gave moderate to high ELISA signals—which we have never seen in MG/thymoma cases [10,11]—but they showed no detectable neutralising activity; both were strongly positive at diagnosis against IFN-α2 and IFN-ω.

Against IL-12, we also found moderate levels of binding autoantibodies in early sera from only five R257X homozygous patients. Only in one of patient did the anti-IL-12 antibodies precede the anti-IFN antibodies (patient A; Table 3), and only in one other did they persist (for ∼6 y).

Against IL-10, granulocyte-macrophage colony-stimulating factor, and tumour necrosis factor–α, we found no significant binding in ELISAs, even though IL-10 is ∼12% homologous to the IFN-λs [17,18]. In numerous sera from many infectious, autoimmune, and neoplastic disorders, we have very rarely found neutralising activity without significant binding (in ELISAs), whereas the reverse is more common [11].

### Potentially Autoimmunising Cell Types

#### In normal thymus.

**Figure 3:**
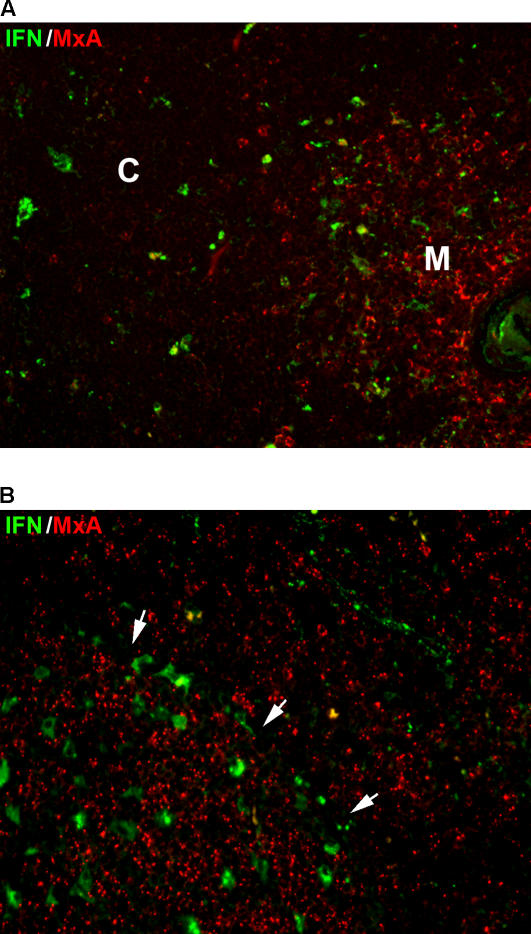
Paraffin Sections of Normal Child Thymus or Reactive Tonsil Double-Labelled for IFN-α and MxA (A) In the thymic cortex (C) to the left, there are scattered IFN-α^+^ cells (green) with almost no nearby MxA^+^ cells (red), but MxA^+^ cells are much more abundant in the medulla (M) to the right, especially around the Hassall's corpuscle at the edge. Results were similar in two other child thymi, though one showed fewer IFN-α^+^ cells in the cortex. The very dense packing precludes precise phenotyping of medullary IFN-α^+^ cells in paraffin sections. (B) Similar staining in a reactive tonsil shows a germinal centre (to the left of the arrows) with several IFN-α^+^ cells and punctate labelling for MxA. Throughout the adjacent T cell area (right), MxA is expressed strongly by a variety of cell types, as already reported [38], whereas IFN-α^+^ cells are concentrated along a small blood vessel.

The similar anti-IFN-α autoantibodies in APS1 and thymoma-associated MG hint at a thymic origin for these responses. To pursue that, we next focussed on the expression and secretion of IFN-α in sections of normal thymus. Essentially all the IFN-α^+^ cells appeared to be CD68^+^ APCs; we saw no significant IFN-α labelling in cytokeratin^+^ cells (not shown). Next, we double-stained for the MxA protein that is up-regulated in cells responding to type I and III IFNs [30]. In the cortex, as expected [31], scattered macrophages (CD68^+^) were strongly IFN-α^+^, but we saw almost no MxA labelling (Figure 3A). By contrast, in the medulla—where *AIRE* is expressed most strongly [4]—there were numerous IFN-α^+^ cells (Figure 3A), and MxA^+^ cells were even more abundant—which is strong evidence of local secretion of type I and III IFNs. Many of the medullary IFN-α^+^ cells were macrophages, and some were almost certainly DCs.

#### In reactive lymph-node-like tissue.

Another striking feature of the anti-IFN autoantibodies in both APS1 and thymoma/MG patients is their prolonged persistence. We uncovered a possible explanation while staining “normal” tonsils (where others have described MxA labelling in extra-follicular areas [38]). The most strongly stained IFN-α^+^ cells proved to be CD68^+^ macrophage-like cells in the germinal centres. Notably, these structures consistently also showed strong to intermediate labelling for MxA (Figure 3B); the reticular pattern of the labelling strongly suggests expression in follicular dendritic cells, and hence suggests that these cells were responding to secreted IFNs. Normally, these highly specialised APCs play a key role in antibody diversification by retaining and displaying native antigens that select and stimulate germinal centre B cells as their antibodies mutate [39]. Thus, any native IFNs being presented there would be ideally situated to perpetuate autoantibody responses against them once initiated (e.g., in a thymoma [20,21]).

#### In APCs cultured from blood.

**Figure 4:**
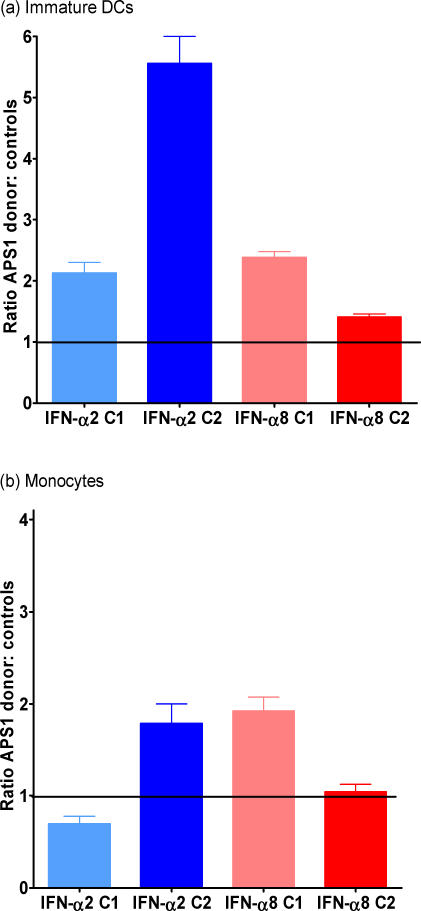
Expression of IFN-α2 and IFN-α8 by Immature DCs and Monocytes Cultured from an APS1 Patient Relative to Two Healthy Blood Donors Expression by immature DCs (A) and monocytes (B). The APS1 donor has typical clinical features of APS1 and high anti–type I IFN titres (see footnote of Table 1). RT-PCR reactions were performed in parallel with *HPRT* as a “house-keeping” gene; *GADPH* gave very similar results when tested in one of the healthy donors, C1 (for IFN-α8). The relative gene (mRNA) expression levels were calculated using the comparative Ct (ΔΔCt) method (according to Applied Biosystems) to yield 2^−ΔΔCt^, where Ct represents the threshold cycle. Every sample was run at least three times in three parallel reactions. Results are depicted in histogram format as ratios of the expression levels in cells from the APS1 donor (which have been given a nominal value of 1.0) to those in the cells from the controls, C1 and C2. The bars represent the standard error of the mean of each ratio.

The above findings imply that the strongest IFN-α production is by APCs, even in the thymus. Since no APS1 thymic cells were available, we compared IFN expression (assessed by RT-PCR) by monocytes and immature and mature DCs cultured from blood from a compound heterozygous APS1 patient (with high anti-IFN-α2 and anti-IFN-ω titres). For IFN-α2 and IFN-α8, the expression levels were consistently higher in the immature DCs from this APS1 patient than from either of two healthy controls (Figure 4A). However, the APS1/control ratios were more variable for the CD14^+^ monocytes (Figure 4B) and mature DCs, and also for IFN-ω in all three cell populations (Table S1).

## Discussion

In two cohorts of Nordic APS1 patients, we have now shown almost 100% prevalences of very high titre IgG neutralising autoantibodies against most IFN-α subtypes and against the related, but antigenically distinct, IFN-ω. However they may be provoked, these precocious, persistent, and highly selective responses against this narrow range of cytokines appear to be APS1-specific and to segregate like a Mendelian recessive trait, regardless of the exact *AIRE* mutations. Their 100% “penetrance” in the homozygotes and compound heterozygotes is notable because APS1 shows such great variability both clinically and serologically [4,7,8,22,23,36]. Moreover, the autoantibodies reached high levels so early that, in almost every informative case, they preceded certain of the autoimmune disorders, and also some of the corresponding endocrine autoantibodies [36]; they even antedated overt CMC, which is usually the earliest sign of APS1. Clearly, these anti-IFN antibody responses cannot be a by-product of any one of these disorders. The converse possibilities—that type I IFNs are involved in autoimmunisation or that the antibodies predispose individuals to CMC—are considered below. For clinicians, our findings together imply a valuable role for these antibodies in diagnosis.

### Clinical Applications

#### In diagnosis.

It now appears that APS1 is even more variable than previously realised; one or more of the characteristic “APS1 triad” (CMC, HP, and AD) may be missing (e.g., Tables 1 and 2)—even in patients with both *AIRE* alleles mutated [22,23]. Since the anti-IFN antibodies show such high prevalences and APS1 specificity, and appear so early and persist so long, they should be valuable for diagnosis of such “incomplete cases” (e.g., the Norwegian brothers discussed in Results), as well as of prodromal APS1 before the appearance of its full clinical picture or of the typical organ-specific autoantibodies. Thus, our data suggest that assays against IFN-α2 and IFN-ω may become the antibody tests of choice, since they were so clearly positive even in patients lacking the other antibodies associated with APS1 [23]. In addition, they were clearly negative in two further clinically doubtful cases with no *AIRE* mutations detected (patients E and F see footnote of Table 3), and in unaffected parents or siblings.

#### In prognosis.

Antibodies against IFN-α2 and IFN-ω might also prove valuable prognostically, since titres clearly can be extremely high even in early childhood. How long or how frequently they precede the onset of APS1 signs is an intriguing question that others may already be able to address.

#### Infections in APS1.

In theory, the anti–type I IFN antibodies might predispose to candidiasis. So might the anti-IL-12 antibodies that we saw early and transiently in a few patients. However, the *AIRE* mutations probably do so more strongly, since this infection is rare in MG/thymoma patients (despite their frequent additional anti-IL-12 antibodies) [11]. If these mutations predispose via parallel pathways to CMC and anti-IFN responses, the latter might hold clues to some even more crucial common defect upstream. If the anti-IFN autoantibodies do contribute to the candidiasis, then cautious treatment with IFN-γ might be considered for intractable cases (with no anti-IFN-γ antibodies); use of IFN-β might carry some risk of autoimmune complications [40]. Recently, Döffinger et al. [41] reported an extraordinary patient (aged 47) with high titre neutralising anti-IFN-γ antibodies who presented with intractable mycobacterial infections (from which he subsequently died). While on IFN-γ therapy (at age ∼51), he developed an APS1-like syndrome including CMC, but no *AIRE* mutations were detected.

The general rarity of recurrent infections in APS1 is a puzzle, in view of (i) the very high neutralising titres against type I IFNs (and IL-12 in MG/thymoma patients), (ii) the role of these IFNs (and of IL-12) in polarising towards pro-inflammatory “T helper 1” T cell responses in humans [15], and (iii) the asplenia in some cases (e.g., Table 3). Any resistance of IFN-αs to neutralisation in vivo might also be relevant clinically, because treatment with anti-IFN-α antibodies has been proposed for certain autoimmune disorders such as systemic lupus erythematosus (SLE), where it is implicated in pathogenesis [42].

Neutralisation of IFN-α evidently can occur in vivo during therapy with IFN-α2; some patients clearly become resistant because they make antibodies against it—usually with much lower titres/cross-reactivity than in APS1 [43,44]. Possibly, however, neutralising antibodies might be less effective in vivo if IFNs secreted in tissues can bind to their receptors before the antibodies neutralise their activity, e.g., because IFN-producing cells are so ubiquitous and mobile or the IFNs they release act at short range. Secondly, many viruses infect mucosal surfaces, where locally produced IFNs may be poorly accessible to circulating neutralising autoantibodies (we find no serum IgA antibodies against IFN-α in MG/thymoma patients; A. Meager, unpublished data). Thirdly, patients with anti-IFN antibodies may instead be protected by IFN-β or IFN-λ—which were neutralised less frequently and mostly at much lower titres. In fact, there are occasional examples of intractable infections in both APS1 and MG/thymoma patients [8,10,11,21]; it may be relevant that one young child with APS1 who had almost continuous respiratory infections also had neutralising titres of 500–2,000 against IFN-β by age 7–9 y. Since MG/thymomas mostly arise in adulthood, we argued [45] that such patients are well-endowed in advance with memory T helper 1 cells that can protect against familiar infections, even during subsequent immunosuppressive therapy. That seems less likely in APS1, where onset is often so much earlier, but where the IL-12→IFN-γ axis may well be compensating.

### Comparing Findings in APS1 and MG/Thymoma Patients

Both the prevalences and the titres of the anti–type I IFN autoantibodies are higher in APS1 patients than in MG/thymoma patients [10,11]; they are also far higher—and much broader in IFN-α subtype cross-reactivity—than those of antibodies induced by therapy with IFN-α [43,44]. There are many provocative parallels with the autoantibodies in MG/thymoma patients [10,11]: their high titres at diagnosis against a similar range of IFN-α subtypes, their protracted persistence—despite immunosuppressive therapy for the MG ([11]; Figure 1A), and the constancy of their IFN-α subtype preferences ([11]; Figure 2).

Among the differences between these two conditions, the hitherto unrecognised autoantibodies to the IFN-λs were barely detectable in MG/thymoma patients, even those positive against IFN-β (A. Meager, unpublished data). In marked contrast, anti-IL-12 antibodies were much more common in MG/thymoma patients [10,11,45] than in patients with APS1, where their very early transient appearance in a few patients suggests that they might be more prevalent before the onset of APS1.

### Clues to Autoimmunising Mechanisms

There is clearly a hierarchy of cytokines recognised in APS1, ranging from IFN-ω (100%) to IFN-α8, to IFN-α2, to IFN-β and IFN-λ1/IFN-λ2, to IL-12 and IFN-γ (<5%). Does that ranking reflect the cell types producing these cytokines, the amounts produced, the stimuli that evoke them, or their immunogenicity or antigenicity? Are the responses against them initiated in the thymus or in the periphery, where *AIRE* is also expressed [2–5]? Are type I IFNs involved in organ-specific autoimmunisation in APS1? The answers to these questions must hold valuable new clues to autoimmunising mechanisms, which are very hard to study in humans. Other clues include (i) the occurrence of anti-IFN-λ1 autoantibodies even in two of ten *AIRE* heterozygotes—which recalls the recently reported gene dosage effects on thymic deletion in *Aire* ^+/−^ mice [46]; (ii) that type I IFN therapy (e.g., for hepatitis C) sometimes precedes a variety of autoimmune disorders [40], including MG, SLE, sarcoidosis, and thyroid disease [47,48]; (iii) that aberrant IFN-α production is well known in SLE [42,49], where sporadic anti-IFN-α autoantibodies have also been noted [50], some of which prefer IFN-α8 (D. Isenberg and A. Meager, unpublished data); and (iv) that autoimmunisation apparently occurs within thymomas ([20,21]; see below).

The anti–type I IFN autoantibodies might be evoked by changes in the cells producing IFNs—which include potent APCs. For example, DCs that produce IFN-α and IFN-β are strongly implicated in autoimmunisation in psoriasis and type 1 diabetes (reviewed in [51]) as well as SLE [49]. It may be relevant that we found increased expression—particularly of IFN-α2 and IFN-8—in blood-derived immature DCs in one APS1 patient (Figure 4A). Because of the variability in expression levels (especially for IFN-ω and in mature DCs), these initial experiments demand to be extended to larger numbers of patients and controls, and above all to cells from thymus (both APCs and MECs). The higher transcript levels in the APS1 patient than the controls (Figure 4A) already suggest that *AIRE* can have inhibitory effects. In fact, these are now well recognised, and extend to many other genes, such as major histocompatibility complex class II genes, and to macrophages [52] as well as MECs [53].

Possibly, in APS1, there might be dysregulated surges in IFN secretion in vivo, e.g., in response to childhood infections such as *Varicella;* indeed, we also find modest neutralising titres against IFN-αs, IFN-ω, and IFN-λ1 (but not IFN-β or IFN-γ) in some hyperimmune anti-*Varicella* Ig preparations from healthy donors ([54]; A. Meager, unpublished data). Furthermore, type I IFNs can themselves act as pro-inflammatory “danger” signals [49] and activate APCs [55]—and thus play a very important role in linking innate and adaptive immune responses. Notably, too, a very early change in newly diabetic pancreatic islets is a striking and sometimes long-lasting up-regulation of IFN-α expression in the β cells [32,56]. In theory, that might help to autoimmunise against islet cell antigens; however, we have found no autoantibodies against IFN-α or IFN-ω in children with sporadic type I diabetes (or in adults with thyroid disease) [11].

It is tempting to look in the thymus for links between APS1 and thymomas; our evidence of autoimmunisation in these tumours [20,21] implies that expression of peripheral self-antigens there is “dangerous”, possibly because of pro-inflammatory signals from dying cells [57] or natural killer cells [58] that could activate DCs and invoke innate as well as adaptive responses. Since they are clearly expressed and secreted in the normal thymic medulla (Figure 3), type I IFNs might, in theory, be involved in the normal induction of tolerance there to peripheral self-antigens, or in the early stages of autoimmunisation against them in APS1. If so, the abnormalities in this process in *AIRE*-mutant patients—or excessive cell death—might somehow lead to autoimmunisation against autoantigens such as type I IFNs that might be even more abundant in the APS1 than the normal thymus; the above hierarchy might be a valuable clue to help to identify the cell types responsible. At a later stage, the expression—and evident secretion—of IFN-α in germinal centres (key sites of B cell clonal diversification/memory generation [39]) might help to perpetuate the autoantibody response against it, and so explain its remarkably prolonged persistence. It might also contribute to the induction of IFN regulatory factor–8 there, which plays an important role in germinal centre physiology [59].

In conclusion, we demonstrate early and persistent prevalence in APS1 patients of apparently spontaneous neutralising autoantibodies to type I IFNs, responses that behave like a Mendelian trait. Such high “penetrance” is very unusual, even in inbred mice, and may have practical implications for clinicians as well as researchers. Because they are so APS1-specific and so consistent in this very variable condition, the autoantibodies against IFN-α and especially IFN-ω should be valuable diagnostically, e.g., to identify atypical or prodromal APS1 in patients with isolated candidiasis, AD, or HP [23], and perhaps also for prognosis, if they subsequently prove to predict onset time of the disease. Finally, if the autoantibodies predispose to candidiasis, this condition might yield to treatment with IFN-γ, which was rarely recognised by APS1 sera.

## Supporting Information

Table S1 Expression of IFN-α2, IFN-α8, and IFN-ω by Monocytes and Immature and Mature DCs Cultured from an APS1 Patient and Healthy Blood Donors (50 KB DOC)

## Abbreviations

AD: Addison disease
*AIRE*: *autoimmune regulator*
APC: antigen-presenting cell
APS[number]: autoimmune polyendocrinopathy syndrome type [number]
AVINA: antiviral interferon neutralisation assay
CMC: chronic muco-cutaneous candidiasis
DC: dendritic cell
ELISA: enzyme-linked immunoabsorbent assay
HP: hypoparathyroidism
IFN: interferon
Ig: immunoglobulin
IL: interleukin
LU: laboratory units
MEC: medullary epithelial cell
MG: myasthenia gravis
RT-PCR: real-time PCR
SLE: systemic lupus erythematosus
