# Supplementary material for: Anti-Interferon Autoantibodies in Autoimmune Polyendocrinopathy Syndrome Type 1
Source: PLoS Med. 2006 Jun 13;3(7):e289. doi: 10.1371/journal.pmed.0030289 (PMC1475653; doi:10.1371/journal.pmed.0030289)
Supplement: Supplementary file 1 [file pmed.0030289.st001.doc]

**Table S1. Expression of IFN-α2, IFN-α8 and IFN-ω by monocytes and immature and mature DCs cultured from an APS1 patient and healthy blood donors**

| **Cells 🡪** | CD14+ | | | | | Immature DC | | | | | Mature DC | | | | |
| --- | --- | --- | --- | --- | --- | --- | --- | --- | --- | --- | --- | --- | --- | --- | --- |
| **Donor 🡪** | APS1 | Contr1 | Ratio1  APS1/  Contr1 | Contr2 | Ratio2  APS1/  Contr2 | APS1 | Contr1 | Ratio1  APS1/  Contr1 | Contr2 | Ratio2  APS1/  Contr2 | APS1 | Contr1 | Ratio1  APS1/  Contr1 | Contr2 | Ratio2  APS1/  Contr2 |
| ← 2^-ΔΔCT^ ± sem → | | | | | | | | | | | | | | | |
| **IFNα2/**  **HPRT** | 1.00  ±.12 | 1.44  ±.08 | **0.69** | 0.56  ±.07 | **1.78** | 1.00  ±.08 | 0.47  ±.11 | **2.12** | 0.18  ±.02 | **5.56** | 1.00  ±.13 | 0.83  ±.13 | **1.20** | 2.94  ±.07 | **0.34** |
| **IFNα8/**  **HPRT** | 1.00  ±.08 | 0.52  ±.02 | **1.92** | 0.96  ±.08 | **1.04** | 1.00  ±.04 | 0.42  ±.04 | **2.38** | 0.71  ±.21 | **1.41** | 1.00  ±.21 | 0.30  ±.03 | **3.33** | 6.0  ±.07 | **0.17** |
| **IFNα8/**  **GAPDH** | 1.00  ±.07 | 0.63  ±.09 | **1.59** |  | **ND** | 1.00  ±.04 | 0.20  ±.04 | **4.91** |  | **ND** | 1.00  ±.03 | 0.38  ±.07 | **2.63** |  | **ND** |
| **IFNω/**  **HPRT** | 1.00  ±.12 | 0.32  ±.04 | **3.1** | 4.17  ±.07 | **0.24** | 1.00  ±.04 | 0.30  ±.05 | **3.32** | 1.82  ±.04 | **0.55** | 1.00  ±04 | 0.24  ±.04 | **4.14** | 1.42  ±.03 | **0.7** |
| **IFNω/**  **GAPDH** | 1.00  ±.10 | 0.37  ±.09 | **2.7** |  | **ND** | 1.00  ±.05 | 0.22  ±.09 | **4.54** |  | **ND** | 1.00  ±.07 | 0.24  ±.10 | **4.16** |  | **ND** |

The APS1 donor has typical clinical features of APS1 and high anti-type I IFN titres (Table 3 legend). Real time PCR reactions were performed in parallel with either HPRT or GAPDH as ‘house-keeping’ genes. Relative transcript levels were calculated using comparative *Ct* (ΔΔ*Ct*) method (according to Applied Biosystems), to yield the 2^-ΔΔCt^ (*Ct* represents the threshold cycle). Every sample was run at least three times in three parallel reactions. Results are expressed as ratios of the expression levels of the APS1 cells (set at 1.0) to each control.
